# On Criminal Responsibility

**Published:** 1856-04-01

**Authors:** Robert Hunter Semple

**Affiliations:** Licentiate of the Royal College of Physicians, Physician to the Northern Dispensary


### ON CRIMINAL RESPONSIBILITY.

BY ROBERT HUNTER SEMPLE, M.D.,

*Licentiate of the Royal College of Physicians, Physician to the Northern Dispensary.*

THE records of our criminal jurisprudence are fertile in instances in which the most atrocious crimes are perpetrated by persons who are absolved from responsibility on the ground of insanity. The mind is horrified at the tale of a mother cutting the throats of her helpless infants as they lie slumbering in their beds, or of another parent hurrying away her progeny in the darkness of the night, and drowning them in the river. These persons are acquitted, and no doubt properly, because they are irresponsible for their actions. But in another case, a man of apparent respectability and good education entertains a groundless suspicion that a clergyman, with whom he had been formerly acquainted, had formed an improper connexion with his wife; and acting upon this delusion, he procures a pistol, balls, and other ammunition, travels down to the village where the clergyman is accustomed to preach, and enters the church with the obvious intention of murdering the preacher; but by a fortunate accident he leaves at the inn the bag containing the loaded pistol, and brings into the church another bag which does not belong to him. Thus his murderous intention is frustrated; but as the abortive attempt is only a sequence or an accompaniment of a series of threats directed at the unoffending clergyman, the latter brings his tormentor to the bar of justice; yet upon a verbal promise to desist from annoyance (a promise which is not kept), and upon his finding bail for his good behaviour, and for his appearance when called upon to receive judgment, the accused is allowed to escape.

I cannot help feeling that cases like the above, which are almost of daily occurrence, deserve the most serious attention, not only of the profession, but of the public at large; and the opportunity is an appropriate one to offer a few remarks upon the difference between Insanity and Crime, upon the practice of the legal profession in the cases now under consideration, and upon the duties of the medical practitioner when placed in relation with insane persons, either before or after their appearance at a criminal bar.

Although it may seem plain enough at first sight to distinguish insanity from crime, the fact is that the boundaries which separate the one from the other are by no means accurately defined, and the difficulties of the subject increase as we proceed in the investigation; and those who are in the habit, like some superficial barristers, of abusing the medical witness because he cannot draw the exact line which divides responsibility from irresponsibility, will find, upon a little investigation, that the difficulty exists in the principles of the law itself, and more deeply still in the very constitution of the human mind. For the aspirations of the law, in its highest development, are directed to the discovery of that abstract principle of equity, which has never yet been isolated, and like the mind itself, has never been disencumbered from the dross around it. The Roman poet says,—

> Sunt certi denique fines
> Quos ultra citraque nequit consistere rectum; but the boundaries are yet to be discovered, and the abstract principle of right is as far away as it was in the time of Augustus.

In the absence of any defined standard of justice, to which all mankind may implicitly submit, the various nations of the earth have erected standards for themselves, and this standard in each country constitutes what is called the law; and as it often happens that what is lawful in one country is unlawful in another, and *vice versâ;* and as, moreover, the law itself is differently interpreted by its own members, it follows, after all, that the limits of right and wrong are determined in different nations upon certain conventional principles, which are more or less conducive to the general welfare and happiness of the human race, in proportion as the nations themselves are more or less advanced in civilization and refinement.

Now, any person who violates the rules thus laid down by a community for the general welfare, becomes a criminal, and amenable to the law. Thus murder, which robs a person of his life, and theft, which robs him of his property, are crimes, because they are clearly injurious to the welfare of the whole people; but that the mere destruction of human life is not a crime in itself, is proved by the fact, that the slaughter of our enemies in war has ever been considered, in the most enlightened countries, rather meritorious than otherwise; and even in the case of theft, the moral guilt of the delinquent is very much modified by a consideration of the motive which has prompted the act. The wretch who steals a quartern loaf to allay the pangs of hunger has a very different moral responsibility from him who habitually steals because he is too idle to work.

If it is so difficult to give an accurate definition of crime, it is still more so to give one of insanity; and especially since the law vaguely defines insanity as the inability to distinguish between right and wrong; whereas neither the lawyers nor any other human beings can accurately define the meaning of these two words. If I were inclined to sophistry, it would not be very difficult to show that crime itself is a species of insanity; for the law having been made for the good of all, whether individuals or communities, it is clear that he who commits a crime is really injuring himself; and as his so doing is contrary to the common instincts of human nature, he ought to be considered insane.

But it is evident that this idea of the identity of crime with insanity can only be regarded as a metaphysical abstraction, and that its introduction into practice would be attended with the most destructive consequences, and, therefore, in the great majority of cases, criminals are considered as responsible beings, and, as such, amenable to the law of the land. But I think it will be readily admitted that the modern treatment of crime is founded upon the view that it is really an insanity, or, rather, an *unsoundness* of the mental faculties; for many of our prisons are not so much abodes of punishment as schools of reformation; and even in the treatment of our worst criminals, a plan of moral and physical indulgence is pursued, which certainly makes the lot of the felon more enviable than that of the pauper. I do not blame this system; I only adduce it as a proof that crime is regarded and treated by our legislators as a species of moral disease.

But, again, there is a class of persons who violate the conventional rules of the community in which they live, or, in other words, commit unlawful acts, but who do so without being conscious that such acts are unlawful. This may happen in three distinct sets of offenders:—1st. Among those who have never received any education, and, therefore, are unable to discern between what, in any community, is considered right and what is considered wrong; 2nd. Among those who are idiotic from birth, and who, from possessing small brains, or from diseased conditions of that organ, are reduced to a level with the brutes; and, 3rd. Among those who, having once received some kind of education, and possessing a well-proportioned cerebral development, fall into what is called insanity, or unsoundness of mind.

It is obvious that the first two sets may be dismissed at once, but the great difficulty lies with the third set, and the great problem to be determined is, how to establish the principles which are to shield the criminal from the consequences of his offence, and to transfer his treatment from the discipline of the gaol, or suspension by the halter, to the Medical superintendence of a lunatic asylum.

Upon this very difficult point I have no dogmatical opinions to offer, because every case must be treated upon its own merits, and no rule can be laid down which can be available in every instance. I repudiate the idea that the solution of the difficulty is to be found in the use of the technical language or of the artificial distinctions drawn by the lawyers, and insisted upon by them with such pertinacity, and I might add, ignorance. I even doubt whether *physical* Medical examinations (except in certain cases, as, for instance, where a woman commits a crime while labouring under puerperal mania) throw very much light upon the matter. The pulse of the accused may be tranquil, the tongue clean, the head cool, the secretions regular, and yet he may have murdered his father, his wife, or his children; nay more, I may state as the result of very considerable experience, that insanity may exist to a high degree without any appreciable lesion of the cerebral organs. I say *appreciable*, because I do not deny that some physical changes may exist in the brain in every case of insanity; I only assert, that I do not know what they are, and that the alleged appearances of congestion of the membranes, subarachnoid effusion, slight degrees of induration or softening of the cerebral substance, all may exist in cases where there is no insanity at all, or may be wanting in cases of well-marked and long-continued aberration of intellect. Without at all denying that the brain is the instrument of the mind, I believe that we are still very far from knowing the exact relation which exists between the structure of the one and the functions of the other; and, in the mean time, I attach little confidence to the attempts which have been made from time to time to explain the wanderings of a morbid fancy by reference to supposed physical appearances.

But although the *physical* Medical examination of a lunatic patient too often throws very little light upon our investigations, the *psychical* Medical examination is a most important and indispensable part of our duties; and apart from the perplexing technicalities of modern lawyers, may and often does lead to correct conclusions with regard to the responsibility or otherwise of the accused. Here we must rejoice that the studies which fit us for our profession enable us to pass the boundaries of matter, and to explore the mysteries of the spiritual world; and difficult as the task undoubtedly is, we must seek by a study of the anatomy (so to speak) of the human mind, for a clue to the morbid conditions of the same powerful principle. We must examine the general character and disposition of the patient; must ascertain the degree of education he has received; must trace the relative importance of his intellectual, moral, or merely animal faculties; must dive as far as possible into his principles of thought and action; must ascertain whether any remarkable change has lately, or at any time, changed or perverted the current of his ideas or of his conduct.

Now, the practical result which I wish to draw from the above remarks is, the improvement of the present practice in relation to what is called criminal lunacy. In the cases alluded to at the commencement of this paper, crimes of the most atrocious character,—murders, by mothers, of their own offspring,—have been undoubtedly committed; and during the trial of both these unhappy women, evidence of a most convincing character was adduced to prove the irresponsibility of the murderesses. But the question naturally arises, if the evidence was so convincing of insanity, *after* the commission of murder, why did no one take the trouble of pointing out the facts *before* human life was so cruelly sacrificed? In one case, it was proved that the prisoner had suffered from milk fever (probably, puerperal insanity), and that her mind had never recovered its tone; and that, moreover, her father was confined in an asylum as an incurable lunatic; in the other case, it was shown that the mother conceived both herself and her children to be labouring under incurable diseases, and that she destroyed them as the most merciful plan of terminating their miseries. Now, can any one for a moment doubt that in both these cases there existed obvious and dangerous delusions? Why did not some of the witnesses, and especially the Medical ones, keep a watchful eye upon the conduct of these unhappy mothers, and thus prevent the commission of horrible infanticide? Why is the commission of murder to be the only valid evidence of insanity?

We believe the answer is to be found in the feeling of the present day, which is almost a morbid feeling,—to allow liberty to every one to the fullest extent, even at the risk of allowing injury to be inflicted on others. I am very far, indeed, from wishing to infringe in any manner upon the great and glorious privilege which belongs to the British subject, of being the uncontrolled master of his own actions; but I maintain, that when the mind of a man falls into a morbid state, such as may endanger the safety and welfare of the community, such a person should be, not punished, but controlled from injuring himself or others. That the present state of the law is culpably lax, by carrying a principle, good in itself, to too great a length, is abundantly evident from the other case to which I have directed attention; where a man, entertaining a *groundless* suspicion as to the virtue of his wife, utters a series of menaces against the *supposed* adulterer, and follows up his threats by an actual attempt (happily abortive) to destroy an innocent man. If the latter *had* been destroyed, the assassin would of course have been acquitted on the ground of insanity; and yet, because he has not *succeeded* in his murderous attempt, he is allowed to go loose, upon his verbal promise—the promise of a lunatic!

I am quite aware of the difficulty of the subject now introduced; other and abler pens than mine may probably enlarge upon a topic which is fertile in interest to all civilized communities; but my conclusion is, that the law should, if possible, *prevent* lunatics from committing crimes, instead of confining them in a lunatic asylum *after* the crimes have been committed. In the case of the person who attempted to shoot the clergyman, the evidence of insanity is so clear as ought to have rendered it imperative upon the Judge to detain the accused, and place him under medical care; but there are several milder forms of mental aberration which no less require the vigilance of relatives. During last week, a gentleman entered the boxes of a crowded theatre, in a state of intoxication, armed with a revolving pistol (fortunately not loaded), which he pointed at the audience. Can there be any doubt that this person's conduct was of a dangerous tendency? could he have any reason to complain, if, acting like a madman, he should, at least for the time, be treated like one?

What I would therefore urge is, that the whole subject of criminal lunacy should be carefully reconsidered by our statesmen, our lawyers, *and the members of our profession;* and I say emphatically, that the latter should have a principal share in the deliberations. I have no taste for *dilettanti* noblemen, however amiable and excellent they may be, usurping to themselves the right of exclusive legislation upon a subject which belongs to our profession, and in which our opinions ought to have very great weight. Nor have I any profound regard for legal decisions coming from Judges, who, in maintaining (and properly, within due limits) the liberty of the subject, would allow of the commission of crime.

I maintain that in cases where delusions are proved to exist, and more especially where those delusions may lead to actions dangerous to society, a degree of *surveillance* ought to be exercised. If a man either is a madman, or pretends to act like one, it can do him no harm whatever that his actions should be watched; and even if it should turn out that his malady is temporary, or that his conduct was merely assumed, I cannot understand why he should be otherwise than grateful to those who have kindly interposed to prevent anticipated evil. I do not urge that all such cases should be locked up in lunatic asylums, but I do maintain that they should be carefully attended by competent persons, until their sanity or insanity is completely established. A person who is suspected of picking a pocket, and is taken to the station-house, has no redress if he were not confined from malice, but upon reasonable suspicion; why should a supposed insane person complain, if, in mercy to himself, he is prevented, by gentle *surveillance*, from hurting himself or society.

It is impossible to exaggerate the evils consequent upon the present practice, which is founded upon a mistaken view of personal rights, and upon the decrees, often arbitrary and often changing, of legal authorities; but the instances have now become so numerous that they are likely to attract attention. Every one must rejoice that the treatment of insanity is founded in the present day upon humane and enlightened principles; but let not this proper relaxation in the severity formerly exercised towards lunatics, lead us into the opposite extreme, and expose the lives of helpless infants, or of other unoffending members of society, to the random attacks of irresponsible assassins, who are proper objects for the vigilance of the State.—*Medical Times and Gazette.*

